# Amino Acid Profiling with Chemometric Analysis as a Feasible Tool for the Discrimination of Marine-Derived Peptide Powders

**DOI:** 10.3390/foods10061294

**Published:** 2021-06-04

**Authors:** Qin Wang, Yanchao Wang, Xiaoming Jiang, Lei Ma, Zhaojie Li, Yaoguang Chang, Yuming Wang, Changhu Xue

**Affiliations:** 1 College of Food Science and Engineering, Ocean University of China, Qingdao 266003, China; wangq218@126.com (Q.W.); wangyanchao@ouc.edu.cn (Y.W.); jxm@ouc.edu.cn (X.J.); malei@ouc.edu.cn (L.M.); lizhaojie@ouc.edu.cn (Z.L.); changyg@ouc.edu.cn (Y.C.); wangyuming@ouc.edu.cn (Y.W.); 2 Laboratory for Marine Drugs and Bioproducts, Qingdao National Laboratory for Marine Science and Technology, Qingdao 266237, China; 3 Qingdao Institute of Marine Bioresources for Nutrition & Health Innovation, Qingdao 266109, China

**Keywords:** marine-derived peptides, amino acid, chemometric analysis, classification, adulteration detection

## Abstract

Marine-derived peptide powders have suffered from adulteration via the substitution of lower-price peptides or the addition of adulterants in the market. This study aims to establish an effective approach for the discrimination and detection of adulterants for four representative categories of marine-derived peptide powders, namely, oyster peptides, sea cucumber peptides, Antarctic krill peptides, and fish skin peptides, based on amino acid profiling alongside chemometric analysis. The principal component analysis and orthogonal partial least squares discriminant analysis results indicate that four categories of marine-derived peptides could be distinctly classified into four clusters and aggregated with the respective raw materials. Taurine, glycine, lysine, and protein contents were the major discriminants. A reliable classification model was constructed and validated by the prediction dataset, mixture sample dataset, and unclassified sample dataset with accuracy values of 100%, 100%, and 100%, respectively.

## 1. Introduction

Marine-derived peptide powders are generally manufactured from marine organisms through protease enzymatic hydrolysis [1,2]. Such peptide powders, mainly containing polypeptides, oligopeptides, and amino acids, are derived from protein degradation and belong to a single complex protein hydrolysate product [2,3]. It is generally accepted that peptides are usually easier to absorb than intact proteins due to the lower molecular weight of peptides [3]. Moreover, marine-derived peptides have demonstrated various biological activities, such as antioxidant, anti-inflammatory, anti-fatigue, anti-hypertensive, and anti-obesity activities [4,5,6]. Consequently, marine-derived peptides are attracting more and more attention and have been widely applied in the food, pharmaceutical, and cosmetic industries [1,7].

Echinodermata, Mollusca, Arthropoda, and Chordata, representing typical species of edible marine animals, have been widely used to produce marine-derived peptide powders. Especially, four categories of marine-derived peptides, namely, sea cucumber peptides, oyster peptides, Antarctic krill peptides, and fish skin peptides, occupy considerable shares in the marine-derived peptide market. The biological activity claims and prices of marine-derived peptide powders differ largely from each other depending on the material categories. In addition, a large variety of animals, plants, and microorganisms from both marine and non-marine origins are involved in the manufacturing of commercial peptide powder products, such as bull backstrap peptides, soybean peptides, and yeast peptides. Since marine-derived peptides generally appear as white or yellow powders, they are often virtually indistinguishable in terms of appearance and this makes them susceptible to adulteration and fraud. There are many possible types of adulteration during the manufacturing process of peptide powder products. The common practice of adulteration includes a simple reduction of purity by an excessive use of food ingredients presented in the preparation process, such as maltodextrin. Similar to other powder products, marine-derived peptide powders have also become the target of adulteration with the deliberate addition of inexpensive constituents, such as gelatin. In addition, a more sophisticated form of adulteration is the substitution of similar peptides which are hydrolyzed by other cheaper materials. It is imperative and meaningful to develop a methodology to discriminate different types of marine-derived peptide powders for the purpose of adulteration detection.

Many analytical techniques have been employed for food fraud and adulteration, including molecular, chromatographic, spectroscopic, mass spectrometric, and electrochemical approaches [8,9,10]. DNA-based techniques are among the most effective methods for the verification of fish and seafood authenticity and the detection of adulteration [8,11,12]. Moreover, proteomic, metabolomic, and lipidomic approaches have recently been examined as potential methods to detect the adulteration of different fish species and other aquatic products [12]. Current research of marine-derived protein hydrolysates has mainly been focused on functional properties, preparation processes, applications, and storage technologies [1,7,13,14,15]; however, there have been few published studies regarding the adulteration detection of marine-derived peptide powders with the substitution of similar peptides from other materials.

Proteins and peptides are comprised of different types of amino acids, indicating that amino acid composition is one of the most important characteristics for marine-derived peptide powders. Many studies have shown that amino acid profiles are selected as potential fingerprints for adulteration detection or classification of different types of food, most often by means of multivariate statistical analysis. For example, chemometric analyses of amino acid profiles have exhibited good classification for most fruit types and this has been further applied in the detection of fruit juice adulterations [16,17,18,19]. Vegetable oils and honey samples could be correctly classified according to their botanical origins or geographic regions based on amino acid profiles with chemometric approaches [20,21,22,23,24]. Amino acid profiles have also been proven to be potential markers for the origin assessment of Serra da Estrela cheese and authenticity identification of plastron-derived functional foods [25,26]. Here, the objective of this study is to determine characteristic amino acids profiles for four categories of marine-derived peptide powders, namely, sea cucumber peptides, oyster peptides, Antarctic krill peptides, and fish skin peptides, and investigate the feasibility of employing amino acid profiles alongside chemometric analysis for the detection of adulteration. Multivariate statistical analysis, including principal component analysis (PCA), orthogonal partial least square discriminant analysis (OPLS-DA), and soft independent modelling of class analogy (SIMCA), namely PCA-Class, are employed for the establishment and verification of classification models.

## 2. Materials and Methods

### 2.1. Sample Collection and Preparation

#### 2.1.1. Raw Marine Material Preparation

For the raw marine material, 4 categories of marine raw materials were purchased from reliable local suppliers, including 12 oyster meat samples from 4 geographical regions in China, 6 fish skin samples from 2 species, 12 sea cucumber samples, covering 5 species and 6 processing techniques, and 12 Antarctic krill samples involving 4 processing techniques. These 42 raw marine material samples were coded according to the given category and detailed sample information is listed in Supplementary Table S1. Antarctic krill meal samples were used for analysis without pre-treatment, and the remaining raw material samples were frozen in liquid nitrogen and then ground into powder. All the raw material samples were stored at −20 °C for further analysis.

#### 2.1.2. Marine-Derived Peptide Powder Preparation

A total number of 66 marine-derived peptide powder samples, including 18 oyster peptides (OP), 16 sea cucumber peptides (SCP), 18 Antarctic krill peptides (AKP), and 14 fish skin peptides (FSP) were collected. These marine-derived peptide powder samples were mostly purchased from reputable and qualified companies in Chinese markets. Due to the limit amounts of reliable suppliers, five OP and eight SCP samples were self-prepared through enzymatic hydrolysis in our laboratory. All peptide powder samples were stored in a desiccator at room temperature and used for analysis directly without pre-treatment.

#### 2.1.3. In Silico Marine-Derived Peptide Mixture Preparation

The marine-derived peptide powders mentioned above were randomly selected to prepare the following seven sets of mixture peptide samples, including four sets of binary mixture samples (M1, SCP:OP = 1:1; M2, AKP:OP = 1:1; M3, SCP:AKP = 1:1; M4, SCP:FSP = 1:1), two sets of ternary samples (M5, SCP:OP:AKP = 1:1:1; M6, FSP:OP:AKP = 1:1:1), and one set of quaternary samples (M7, SCP:OP:AKP:FSP = 1:1:1:1). Each set contained four mixture samples and the amino acid composition data of 28 mixture samples were obtained by in silico calculation according to the amino acid composition of respective samples obtained in Section 2.1.2. The amino acid data of the 28 in silico mixture samples were used for further analysis.

#### 2.1.4. Unclassified Peptide Powder Preparation

Nine peptide powders from other categories of marine and non-marine origins, involving abalone peptides, octopus peptides, crocodile peptides, cuttlefish peptides, bull backstrap peptides, and donkey hide gelatin peptides, were self-prepared and assembled into an unclassified dataset for application of the classification models.

### 2.2. Moisture and Protein Content Determination

Moisture content was determined by drying in the oven at 105 °C until a constant weight was obtained according to the standard AOAC method [27]. The crude protein content was determined using an automatic Kjeldahl nitrogen analyzer (KjeltecTM8400, FOSS Quality Assurance Co., Ltd., Copenhagen, Denmark) according to the AOAC method [27]. The conversion factor of 6.25 was used to calculate the crude protein contents for all the samples. The crude protein content was expressed on a percentage of dry weight basis.

### 2.3. Amino Acid Profile Analysis

Amino acid compositions were analyzed by an automatic amino acid analyzer (L-8900, Hitachi Global Co., Ltd., Tokyo, Japan) following the method described by Cao et al. with some small modifications [28]. Briefly, samples were hydrolyzed with 6 M HCl containing 0.5% 2-mercaptoethanol at 110 °C for 22 h. Following hydrolysis, the sample was evaporated with nitrogen blowing at 50 °C to remove HCl. The residual was dissolved in 0.02 M HCl and then passed through a 0.22-μm membrane filter before injection into the amino acid analyzer. The content of each amino acid was expressed as g/100 g dry protein.

### 2.4. Statistical Analysis

Experimental data were subjected to one-way analysis of variance (ANOVA) by the SPSS 17.0 software package (SPSS Inc., Chicago, IL, USA). Duncan’s test was used to determine significant differences between samples (*p* < 0.05). Chemometric analysis was performed to discriminate peptide samples in different categories using the SIMCA software package (Sartorius, Malmö, Sweden). Variable importance in projection (VIP) analysis was used to find the most influential variables for classification [29]. A PCA-Class was used to construct the classification model for each marine-derived peptide sample category. The collected data of 108 samples, including raw materials and marine derived peptides, were randomly split into a training dataset and a prediction dataset in a ratio of 2:1, respectively. The prediction dataset consisted of 36 samples from these 4 categories, including raw materials (*n* = 12) and their derived peptides (*n* = 24), while the remaining 72 samples were used to build the training dataset. The Mahalanobis distance (DModX PS+) was used to detect outliers in the four PCA-Class submodels. The sample in which the Mahalanobis distance was larger than Dcrit (95% confidence interval) of certain submodel was considered an outlier [30]. Accuracy was used to determine the PCA-Class model’s classification performance, which was defined as the proportion of correctly classified samples to total samples.

## 3. Results and Discussion

### 3.1. Classification of Four Categories of Raw Marine Materials

Table 1 lists the amino acid compositions and average crude protein contents of the four marine material categories, including the oyster, Antarctic krill, sea cucumber, and fish skin samples. The oyster samples contained significantly lower protein contents in comparison to other three categories of samples (*p* < 0.05), while the fish skin samples contained significantly higher protein contents than the other three categories of samples (*p* < 0.05). From the perspective of amino acid composition, the four categories of raw materials contained abundant amounts of GLU, which represented the amino acid with the highest content both in the oyster and Antarctic krill groups. The oyster group was detected with a high level of TAU, while TAU was present in very small amounts in the other three categories. The amino acid with the highest content in the sea cucumber and fish skin groups was GLY. The sea cucumber and fish skin samples also contained a higher level of PRO than the oyster and Antarctic krill samples.

Linehan et al. reported that the dry protein contents of Pacific oysters (*Crassostrea Gigas*) range from 39.1% to 53.1% due to seasonal variation [31]. In the present study, the significantly lower crude protein contents and a high amount of GLU, ASP, and TAU in oyster meat samples show good agreement with the previous study [32]. Chen et al. determined that whole Antarctic krill (*Euphausia Superba*) contained 76.5% of crude protein on dry weight basis, and that GLU was the amino acid with the highest content after ASP [33]. Wen et al. found that the protein contents of eight commercially processed sea cucumber species ranged from 40.7% to 63.3% and that the most abundant amino acids in sea cucumbers were GLY, GLU, ASP, ALA, and ARG [34]. The high levels of GLY in sea cucumber and fish skin samples were related to the presence of high amounts of collagens, which generally contain a special structure with the sequence of “GLY-X-Y” [35,36]. The differing protein contents and amino acid distribution profiles for these four marine material categories are in accordance with the previously reported results.

**Figure 1:**
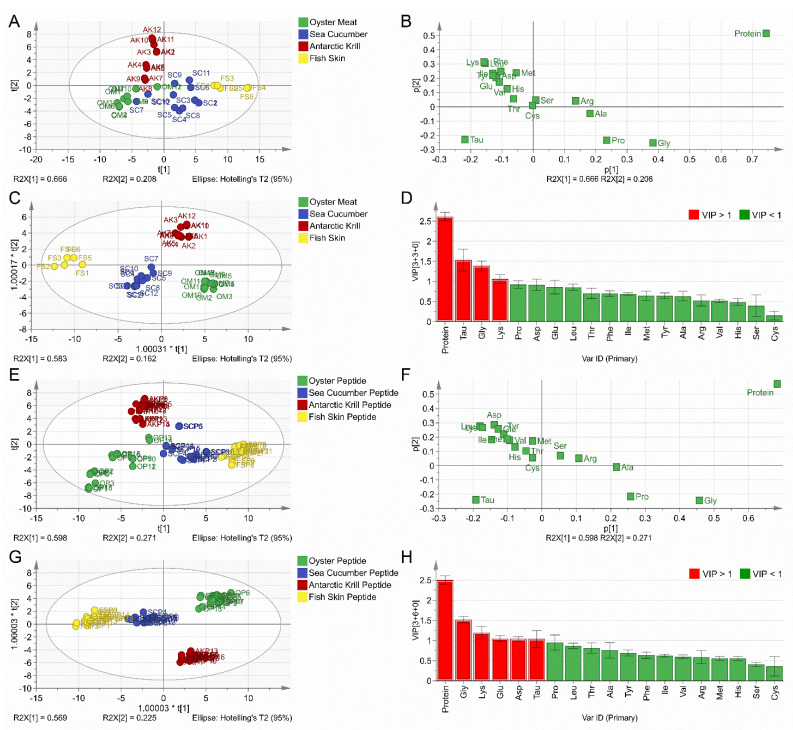
Chemometric analysis. (**A**) Score plot of the PCA for raw materials. (**B**) Loading plot of the PCA for raw materials. (**C**) Score plot of the OPLS–DA for raw materials. (**D**) VIP plot of the OPLS–DA for raw materials. (**E**) Score plot of the PCA for marine-derived peptides. (**F**) Loading plot of the PCA for marine-derived peptides. (**G**) Score plot of the OPLS–DA for marine-derived peptides. (**H**) VIP plot of the OPLS–DA for marine-derived peptides.

The contents of protein and 18 types of amino acids were included in the multivariate statistical analysis for both the PCA and OPLS-DA for the 42 raw material samples. According to the score plot of the PCA model (Figure 1A), the first two principal components (PC1 and PC2) explain 87.40% of the total variance with an R2X (cum) value of 0.991 and a Q2 (cum) value of 0.917. The score plot could be divided into four regions without a clear boundary according to samples in the four categories (Figure 1A). Sea cucumber samples, with the codes of SC7 and SC10, were close to the oyster meat samples in the score plot, which might result from the low protein contents of SC7 (45.79 ± 0.94%) and SC10 (54.27 ± 0.07%). Among all the tested samples, the oyster group showed an average protein content of 49.41 ± 11.74%, while the sea cucumber group contained an average protein content of 72.06 ± 12.22%. The Antarctic krill samples (AK7, AK8, and AK9) were also close to the oyster meat samples due to the lower protein content (64.22 ± 0.40%, 64.22 ± 0.40%, and 64.22 ± 0.40%) than the average protein content (73.56 ± 7.78%) of the Antarctic krill group. The PCA model was unable to provide good clustering among the four categories of marine materials. The loading plot (Figure 1B) showed a strong negative correlation between TAU content and the protein contents for both the PC1 and PC2 scales. With a negative loading score, TAU was the amino acid that accounted for the discrimination of oyster meat samples, while GLY and PRO, with high positive loading scores, were the amino acids that accounted for the discrimination of sea cucumber and fish skin samples into different clusters by PC1. The results of the loading plot are consistent with the data shown in Table 1.

A supervised OPLS-DA model was further employed to achieve more significant clustering and reveal the major variables. It can be seen that the four raw marine material categories may be distinctly classified into four clusters (Figure 1C). In particular, the fish skin and sea cucumber groups were distributed at the first principal component, t[1], with negative coordinates, while the oyster and Antarctic krill groups appeared at t[1] with positive coordinates. Moreover, the latter two groups could be further discriminated along the second principal component, t[2]. The oyster group was projected along with t[2] with negative coordinates, while the Antarctic krill group was distributed along t[2] with positive coordinates. The varying sample distribution profiles could be attributed to the different protein compositions. Previous studies have reported that myofibrillar and sarcoplasmic proteins represent the dominant proteins in oyster and Antarctic krill meat samples, resulting in similar compositions of amino acids from muscle proteins [37,38]. Saito et al. reported that the collagen content was estimated to be about 70% of the total protein in the sea cucumber body wall [35]. The collagen content of cod skins amounts, on average, to 71.2% on a dry weight basis [36]. The similar high concentrations of collagens in the sea cucumber and fish skin samples led to closer distribution profiles in score plots. A VIP plot was employed to indicate the weightage of each variable in the OPLS-DA model to discriminate different classes successfully. The VIP plot shows that the TAU, GLY, LYS, and protein contents are the major discriminants with VIP values above 1 (Figure 1D). It was not surprising to find that the TAU, GLY, LYS, and protein contents, with great discriminative power, were also the variables recorded with high loading scores in Figure 1B. Accordingly, these four categories of marine material could be clearly divided into four clusters depending on the protein contents and amino acid composition profiles.

### 3.2. Classification of Four Categories of Marine-Derived Peptide Powders

As shown in Table 2, the OP group contained significantly lower protein contents (*p* < 0.05) and higher TAU contents than the other three groups. The FSP group contained the highest level of protein contents among the four categories of marine-derived peptide powders. The OP and AKP groups contained high levels of GLU and ASP, while the SCP and FSP groups showed high contents of GLY and GLU. The protein contents and amino acid composition profiles of peptide powders were consistent with those of the respective raw material samples.

The protein contents and 18 types of amino acids were included in the multivariate statistical analysis of both PCA and OPLS-DA for the 66 marine-derived peptide samples. The PCA model exhibited good discriminant power with a R2X (cum) value of 0.916 and a Q2 (cum) value of 0.812 (Figure 1E). The first two principal components (PC1 and PC2) explained 86.8% of the total variance. The amino acid compositions and protein contents of the sea cucumber peptide and oyster peptide samples might be influenced by the geographical locations and processing techniques, which resulted in a dispersed scope for the score plot (Figure 1E). The loading plot of the PCA model for marine-derived peptide samples shows high similarity with the raw marine material samples shown in Figure 1B (Figure 1F). Interestingly, the OPLS-DA score plot illustrates that the four categories of marine-derived peptide samples could be clearly discriminated (Figure 1G). The protein content and certain amino acid compositions (GLY, LYS, GLU, ASP, and TAU) represent the significant variables with VIP values > 1 (Figure 1H).

In the present study, the employed peptide samples were derived from raw marine materials covering various species and different pre-processing techniques were used. A good clustering of the four types of peptide samples could be achieved through multivariate statistical analysis depending on their protein contents and amino acid composition profiles. The results obtained from the score and loading plots of peptide powder samples are consistent with those of the raw material samples.

### 3.3. Establishment and Verification of Classification Models for Marine-Derived Peptide Samples

**Figure 2:**
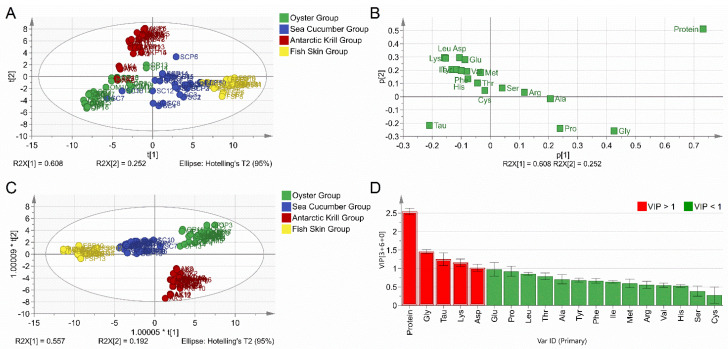
Chemometric analysis for the marine-derived peptides combined with the respective raw materials. (**A**) Score plot of the PCA model. (**B**) Loading plot of the PCA model. (**C**) Score plot of the OPLS–DA model. (**D**) VIP plot of the OPLS–DA model.

To verify the raw material categories of marine-derived peptide powders, the marine-derived peptide samples and corresponding raw material samples were assigned to a new dataset and all 108 samples were divided into four new groups, namely, the oyster group, sea cucumber group, Antarctic krill group, and fish skin group. The contents of protein and 18 types of amino acids were included in the multivariate statistical analysis of both the PCA and OPLS-DA models (Figure 2). The first two principal components (PC1 and PC2) explain 86% of the total variance, with a R2X (cum) value of 0.912 and a Q2 (cum) value of 0.811 (Figure 2A). The results of the loading plot were in line with those of the four categories of raw marine materials and their derived peptide samples (Figure 2B). The four categories of marine-derived peptide samples were clearly classified into four clusters together with their corresponding raw material samples by the OPLS-DA model (Figure 2C). Protein content and certain amino acid compositions (GLY, TAU, LYS, and ASP) were the most important variables that accounted for the discrimination of the four sample categories (Figure 2D).

The PCA and OPLS-DA model exhibited tight cluster formation between peptide samples and respective raw materials and good separation between samples in different categories. The result demonstrates that the marine-derived peptides included in the present study share similar amino acid composition with the respective raw materials, confirming the authenticity of the marine-derived peptide samples. Moreover, the results demonstrate that multivariate statistical analysis is capable of capturing the variance of amino acid compositions between peptide samples from marine materials in different categories, and this further demonstrates the potential of using multivariate statistical analysis for the adulteration assessment of peptide powder samples. This was also shown by Seow et al., who reported that the multivariate statistical analysis of amino acid composition data is an effective method to differentiate between cave and house bird nests [39]. Azevedo et al. determined free amino acid profiles of bracatinga honeydew honey for geographical classification by using a chemometric approach [21]. Botoran et al. ascertained that multivariate statistical analysis, in combination with amino acid profiles, could provide valuable information for the authenticity verification of the varietal origins of fruit juices [19]. Further, Wistaff et al. studied whole amino acid profiles of various fruit types and validated the capability of adulteration detection for blond orange juice added in blood orange juice [18]. As such, multivariate statistical analysis tools, such as PCA and OPLS-DA, when combined with amino acid profiles, provide a feasible and promising strategy for the quality control of marine-derived peptides.

**Figure 3:**
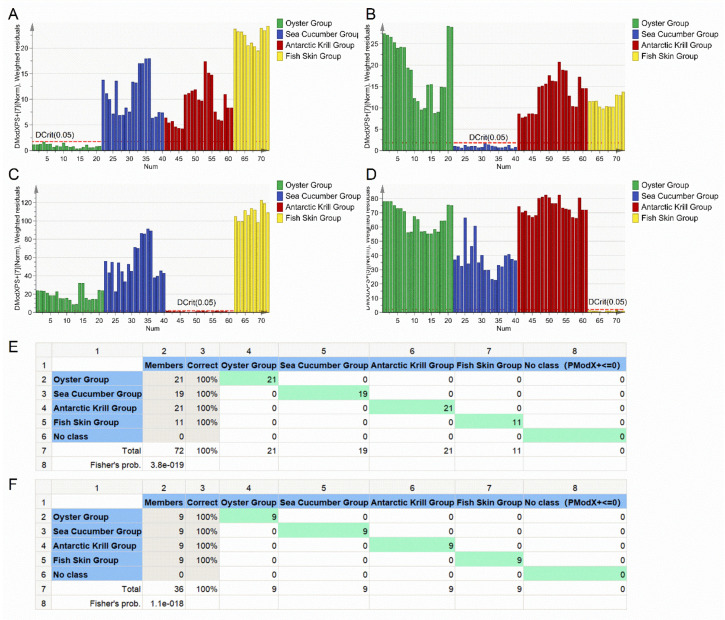
Classification model for the marine-derived peptides. (**A**) DModX PS+ column plot of the oyster group. (**B**) DModX PS+ column plot of the sea cucumber group. (**C**) DModX PS+ column plot of the Antarctic krill group. (**D**) DModX PS+ column plot of the fish skin group. (**E**) Misclassification table of the training set. (**F**) Misclassification table of the prediction set.

A PCA-Class model was applied to establish a classification model for distinguishing between the four categories of marine-derived peptide samples. Four PCA-Class submodels were constructed on basis of the training set, namely the oyster PCA-Class submodel, sea cucumber PCA-Class submodel, Antarctic krill PCA-Class submodel, and fish skin PCA-Class submodel (Figure 3A–D). All samples in the oyster group showed a DModX PS+ < Dcrit (0.05) in the oyster PCA-Class submodel and DModX PS+ > Dcrit (0.05) in the other three submodels. The same phenomenon was observed for samples in the sea cucumber group, Antarctic krill group, and fish skin group with respective submodels (Figure 3A–D). This demonstrates that the four PCA-Class submodels could correctly classify the 72 samples into four groups, indicating that the accuracy of the PCA-Class model was 100% (Figure 3E). A prediction dataset was employed to assess the predictive ability of the constructed classification model as external validation. The four submodels could classify all the 36 samples from the prediction dataset into respective groups with a classification accuracy rate of 100% (Figure 3F). It has been reported that a PCA-Class model built for the authentication of the protected denomination of origin for paprika powder has had an accuracy of 91%, and the PLS-DA model had an accuracy of 96% [30]. These values are lower than those in the present study. The PCA-Class model based on amino acid profiles could be employed to distinguish the raw material categories of marine-derived peptides.

### 3.4. Application of the Classification Model to Adulteration Detection with Marine-Derived Peptide Mixture Samples

**Figure 4:**
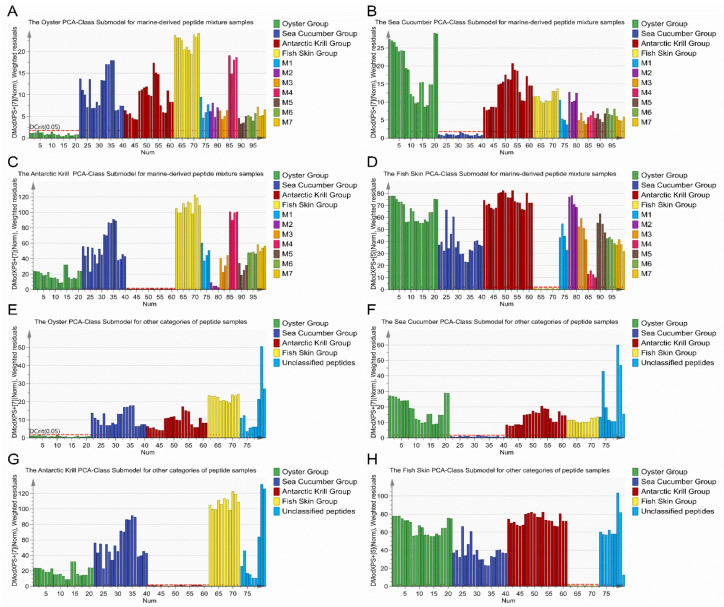
Application of the classification model based on the DModX PS+ column plot for adulteration detection using (**A**–**D**) the marine-derived peptide mixture sample dataset and (**E**–**H**) the unclassified peptide sample dataset.

Considering the fact that marine-derived peptides might be partially substituted with peptides from other marine species, in silico calculation of mixture samples could be employed as a good test to validate the classification model. In addition, the discrimination of mixture samples was a challenge for the classification model because of the high similarity between mixture samples and the modeling samples. In order to increase the variability of mixture samples, binary, ternary, and quaternary peptide mixtures with different combination ratios of the four types of marine-derived peptide powder samples were employed for the application of the classification model to adulteration detection by in silico calculation. The discrimination results of the 28 marine-derived peptide mixture samples are shown in Figure 4A–D. A total of 28 mixture samples showed a DModX PS+ > Dcrit (0.05) in the classification model, indicating that these 28 samples were not classified as belonging to any of the four types of marine-derived peptides; however, one binary mixture sample (M2–4, AKP:OP = 1:1) in Figure 4C was very close to the red line (Dcrit 0.05). Accordingly, the accuracy of classification was 100%. It can be concluded that the classification model built in this study displayed a reasonably good predictive capability with a correct level of 100%, being higher than those previously reported in literature for mixtures of other foods, such as 90.03% to 96.52% for edible oil [40], 93.70% for virgin olive oil [41], and 92.00% to 96.60% for milk [42].

The sample (M2–4, AKP:OP = 1:1) in the present study was a mixture of Antarctic krill peptides and oyster peptides. This seemed to be in line with the high similarity between the Antarctic krill and oyster material in terms of the muscle protein composition. It was hypothesized that extension of the dataset with a larger number of samples to construct models might improve the robustness and accuracy of the model for classification [18]. Overall, the PCA-Class model constructed with limited number of samples in the present study exhibited a clear tendency for the correct classification of marine-derived peptides.

### 3.5. Application of the Classification Model for Adulteration Detection with Other Marine and Non-Marine Peptide Samples

To evaluate the discriminative ability of the constructed classification model for peptide samples from other materials, nine peptide powder samples derived from both marine and non-marine materials were also employed and defined as an unclassified dataset. The amino acid compositions and protein contents of nine unclassified samples are shown in Supplementary Table S2. As presented in Figure 4E–H, all unclassified peptide samples showed a DModX PS+ > Dcrit (0.05) in the four PCA-Class submodels, indicating that the classification accuracy of the model for the nine unclassified peptide samples was 100%. These unclassified peptide samples were correctly differentiated from the four categories of marine-derived peptide powder samples, demonstrating the accuracy and feasibility of the PCA-Class model, based on amino acid profiles, in the classification of marine-derived peptides.

Marine-derived peptides from various materials in different categories have been prepared and applied in the food industry. Furthermore, marine-derived peptides might be produced from different raw materials with the same common name. As an example, sea cucumber peptides might be prepared from different sea cucumber species, such as *Apostichopus japonicus*, *Holotoria floridona*, and *Cucumaria frondosa*. In addition, raw marine materials with different pre-treatment processes, such as fresh sea cucumbers, dried sea cucumbers, and salted sea cucumbers, might be used for the production of peptides. Considering the various material species and production processes of peptide powders, distinguishing between different categories of marine-derived peptides, such as sea cucumber peptides, oyster peptides, krill peptides, and fish skin peptides, seems to be difficult. Inspired by the advancement of chemometric analysis methods, we have established an effective PCA-Class model for the discrimination of marine-derived peptides in different categories based on amino acid profiles, which might be used for the quality control of marine-derived peptide powders in the food industry.

## 4. Conclusions

This study has confirmed the feasibility of discrimination and adulteration detection of four representative categories of marine-derived peptides, including sea cucumber peptides, oyster peptides, Antarctic krill peptides, and fish skin peptides, by employing amino acid profiles combined with chemometric analysis. TAU, GLY, and LYS were concluded to be the major amino acids for the discrimination of the marine-derived peptide powders and the respective raw marine materials by PCA and OPLS-DA models. A PCA-Class model was further applied to construct a classification model for the discrimination of the four categories of marine-derived peptide powders, and the model showed excellent predictive capability with 100% samples being correctly classified in the prediction dataset. The classification accuracy of 28 in silico peptide mixture samples, consisting of binary, ternary, and quaternary peptide mixtures with different combination ratios of the four categories of peptide powders, was 100%. Furthermore, another validation dataset consisting of a total of nine unclassified samples was correctly classified when employing the established PCA-Class model, confirming the robustness of the classification model. The methodology developed in this study seems to be promising and reliable for the classification of the four types of marine-derived peptide powder categories and be suitable for the discrimination of other animal-based peptides with the objective of adulteration detection.

## Figures and Tables

**Table 1:** Crude protein contents and hydrolyzed amino acid compositions of the four raw marine material categories.

Amino Acids ^1^	Amino Acid Content (g/100 g Dry Protein Basis)			
	Oyster Meat (*n* = 12) ^2^	Antarctic Krill (*n* = 12) ^2^	Sea Cucumber (*n* = 12) ^2^	Fish Skin (*n* = 6) ^2^
TAU	7.49 ± 1.42	0.89 ± 0.72	0.06 ± 0.10	0.11 ± 0.12
ASP	7.78 ± 0.79	9.27 ± 1.31	8.19 ± 0.90	5.54 ± 0.60
THR	3.55 ± 0.31	3.83 ± 0.42	4.04 ± 0.36	2.53 ± 0.10
SER	3.65 ± 0.45	3.51 ± 0.50	3.67 ± 0.82	4.06 ± 1.14
GLU	11.33 ± 1.29	12.64 ± 1.78	11.46 ± 1.11	8.88 ± 0.48
GLY	5.10 ± 0.61	4.21 ± 0.18	13.35 ± 2.80	19.09 ± 1.90
ALA	4.72 ± 0.49	5.02 ± 0.51	6.66 ± 1.21	7.97 ± 1.14
CYS	0.21 ± 0.08	0.24 ± 0.04	0.28 ± 0.12	0.20 ± 0.04
VAL	3.41 ± 0.39	4.09 ± 0.33	2.78 ± 0.49	2.13 ± 0.22
MET	1.70 ± 0.14	2.95 ± 0.53	1.10 ± 0.29	1.57 ± 0.33
ILE	3.06 ± 0.22	4.37 ± 0.40	2.26 ± 0.68	1.32 ± 0.37
LEU	5.43 ± 0.43	7.42 ± 0.87	4.01 ± 1.12	3.00 ± 0.36
TYR	2.80 ± 0.50	3.88 ± 0.59	1.92 ± 0.44	0.91 ± 0.32
PHE	2.85 ± 0.25	4.07 ± 0.56	1.73 ± 0.54	1.77 ± 0.06
LYS	5.95 ± 0.64	7.28 ± 1.09	2.56 ± 1.07	3.42 ± 0.39
HIS	1.60 ± 0.17	1.79 ± 0.25	0.81 ± 0.30	0.99 ± 0.26
ARG	5.31 ± 0.70	5.75 ± 0.76	6.75 ± 0.67	7.31 ± 0.58
PRO	4.09 ± 1.09	3.07 ± 0.59	7.15 ± 1.24	9.88 ± 2.07
Total AAs (g/100 g dry protein basis)	80.01 ± 7.77	84.28 ± 8.30	78.76 ± 7.06	80.69 ± 3.24
Crude Protein (g/100 g dry sample basis)	49.41 ± 11.74 ^c^	73.56 ± 7.78 ^b^	72.06 ± 12.22 ^b^	108.94 ± 9.34 ^a^

^1^ Abbreviation: TAU, taurine; ASP, aspartic acid; THR, threonine; SER, serine; GLU, glutamic acid; GLY, glycine; ALA, alanine; CYS, cysteine; VAL, valine; MET, methionine; ILE, isoleucine; LEU, leucine; TYR, tyrosine; PHE, phenylalanine; LYS, lysine; HIS, histidine; ARG, arginine; PRO, proline; total AAs, total amino acids. ^2^ All values are expressed as mean ± standard deviation. ^a,b,c^ Values within the same row sharing different superscript characters are significantly different at a 5% probability level based on ANOVA.

**Table 2:** Crude protein contents and hydrolyzed amino acid compositions of the four marine-derived peptide powder categories.

Amino Acid ^1^	Amino Acid Content (g/100 g Dry Protein Basis)			
	Oyster Peptides	Antarctic Krill Peptides	Sea Cucumber Peptides	Fish Skin Peptides
	(*n* = 18) ^2^	(*n* = 18) ^2^	(*n* = 16) ^2^	(*n* = 14) ^2^
TAU	4.73 ± 2.62	0.38 ± 0.25	0.04 ± 0.14	0.01 ± 0.02
ASP	8.50 ± 1.10	11.57 ± 0.96	9.22 ± 0.91	5.99 ± 0.70
THR	3.68 ± 0.25	4.26 ± 0.65	4.43 ± 0.31	2.90 ± 0.25
SER	3.54 ± 0.33	4.00± 0.60	4.14 ± 0.84	4.35 ± 1.21
GLU	12.77 ± 2.29	15.69 ± 1.44	13.71 ± 1.35	10.27 ± 0.88
GLY	5.89 ± 1.66	4.59 ± 0.26	15.91 ± 3.17	22.94 ± 1.20
ALA	4.88 ± 0.76	6.28 ± 0.45	8.10 ± 1.85	9.18 ± 0.84
CYS	0.17 ± 0.12	0.60 ± 1.04	0.02 ± 0.06	0.07 ± 0.14
VAL	3.56 ± 0.35	4.89 ± 0.23	3.66 ± 0.39	2.66 ± 0.33
MET	1.57 ± 0.21	2.84 ± 0.37	1.44 ± 0.16	1.77 ± 0.29
ILE	3.28 ± 0.43	4.44 ± 0.38	2.39 ± 0.66	1.35 ± 0.09
LEU	5.71 ± 0.69	8.32 ± 0.80	4.19 ± 0.91	2.71 ± 0.43
TYR	2.02 ± 0.35	4.00 ± 0.42	1.96 ± 0.53	0.75 ± 0.09
PHE	2.50 ± 0.46	3.98 ± 0.51	1.82 ± 0.48	1.61 ± 0.17
LYS	6.04 ± 0.63	8.39 ± 0.65	2.58 ± 0.99	3.43 ± 0.49
HIS	1.43 ± 0.20	2.13 ± 0.15	0.78 ± 0.28	0.93 ± 0.10
ARG	5.26 ± 0.56	6.04 ± 1.23	6.85 ± 0.72	6.85 ± 1.54
PRO	4.05 ± 0.94	2.54 ± 0.45	6.74 ± 1.19	10.10 ± 1.71
Total AAs (g/100 g dry protein basis)	79.57 ± 4.93	94.94 ± 5.41	87.98 ± 5.56	87.87 ± 3.74
Crude Protein (g/100 g dry sample basis)	56.97 ± 13.90 ^c^	88.61 ± 3.50 ^b^	86.01 ± 5.71 ^b^	106.36 ± 4.83 ^a^

^1^ Abbreviation: TAU, taurine; ASP, aspartic acid; THR, threonine; SER, serine; GLU, glutamic acid; GLY, glycine; ALA, alanine; CYS, cysteine; VAL, valine; MET, methionine; ILE, isoleucine; LEU, leucine; TYR, tyrosine; PHE, phenylalanine; LYS, lysine; HIS, histidine; ARG, arginine; PRO, proline; total AAs, total amino acids. ^2^ All values are expressed as mean ± standard deviation. ^a,b,c^ Values within the same row sharing different superscript characters are significantly different at a 5% probability level based on ANOVA.

## Data Availability

The data herein presented are available on request from the corresponding author.

## Supplementary Materials

The following are available online at https://www.mdpi.com/article/10.3390/foods10061294/s1. Table S1: Sample information of the raw marine materials. Table S2: Crude protein content and hydrolyzed amino acid composition of nine unclassified peptide samples.

## References

[1] Etemadian Y., Ghaemi V., Shaviklo A.R., Pourashouri P., Mahoonak A.R.S., Rafipour F. (2021). Development of animal/plant-based protein hydrolysate and its application in food, feed and nutraceutical industries: State of the art. J. Cleaner Prod..

[2] Wang Y., Selomulya C. (2020). Spray drying strategy for encapsulation of bioactive peptide powders for food applications. Adv. Powder Technol..

[3] Manninen A.H. (2009). Protein hydrolysates in sports nutrition. Nutr. Metab..

[4] Manikkam V., Vasiljevic T., Donkor O.N., Mathai M.L. (2016). A review of potential marine-derived hypotensive and anti-obesity peptides. Crit. Rev. Food Sci. Nutr..

[5] Ye J., Shen C., Huang Y., Zhang X., Xiao M. (2017). Anti-fatigue activity of sea cucumber peptides prepared from Stichopus japonicus in an endurance swimming rat model. J. Sci. Food Agric..

[6] Qian B., Zhao X., Yang Y., Tian C. (2020). Antioxidant and anti-inflammatory peptide fraction from oyster soft tissue by enzymatic hydrolysis. Food Sci. Nutr..

[7] Pavlicevic M., Maestri E., Marmiroli M. (2020). Marine bioactive peptides-an overview of generation, structure and application with a focus on food sources. Mar. Drugs.

[8] Hong E., Lee S.Y., Jeong J.Y., Park J.M., Kim B.H., Kwon K., Chun H.S. (2017). Modern analytical methods for the detection of food fraud and adulteration by food category. J. Sci. Food Agric..

[9] Medina S., Perestrelo R., Silva P., Pereira J.A.M., Camara J.S. (2019). Current trends and recent advances on food authenticity technologies and chemometric approaches. Trends Food Sci. Technol..

[10] Danezis G.P., Tsagkaris A.S., Camin F., Brusic V., Georgiou C.A. (2016). Food authentication: Techniques, trends & emerging approaches. TrAC Trends Anal. Chem..

[11] Gopi K., Mazumder D., Sammut J., Saintilan N. (2019). Determining the provenance and authenticity of seafood: A review of current methodologies. Trends Food Sci. Technol..

[12] Kotsanopoulos K.V., Exadactylos A., Gkafas G.A., Martsikalis P.V., Parlapani F.F., Boziaris I.S., Arvanitoyannis I.S. (2021). The use of molecular markers in the verification of fish and seafood authenticity and the detection of adulteration. Compr. Rev. Food Sci. Food Saf..

[13] Li X., Yang R., Ju H., Wang K., Lin S. (2021). Identification of dominant spoilage bacteria in sea cucumber protein peptide powders (SCPPs) and methods for controlling the growth of dominant spoilage bacteria by inhibiting hygroscopicity. LWT-Food Sci. Technol..

[14] Li X., Wang K., Yang R., Dong Y., Lin S. (2020). Mechanism of aroma compounds changes from sea cucumber peptide powders (SCPPs) under different storage conditions. Food Res. Int..

[15] Halim N.R.A., Yusof H.M., Sarbon N.M. (2016). Functional and bioactive properties of fish protein hydolysates and peptides: A comprehensive review. Trends Food Sci. Technol..

[16] Tezcan F., Uzasci S., Uyar G., Oztekin N., Erim F.B. (2013). Determination of amino acids in pomegranate juices and fingerprint for adulteration with apple juices. Food Chem..

[17] Asadpoor M., Ansarin M., Nemati M. (2014). Amino Acid profile as a feasible tool for determination of the authenticity of fruit juices. Adv. Pharm. Bull..

[18] Wistaff E.A., Beller S., Schmid A., Neville J.J., Nietner T. (2021). Chemometric analysis of amino acid profiles for detection of fruit juice adulterations—Application to verify authenticity of blood orange juice. Food Chem..

[19] Romina B.O., Elena I.R., Gheorghe M.M., Diana C., Lucian R.G., Raluca P. (2019). Amino acid profile of fruits as potential fingerprints of varietal origin. Molecules.

[20] Rebane R., Herodes K. (2008). Evaluation of the Botanical Origin of Estonian Uni- and Polyfloral Honeys by Amino Acid Content. J. Agric. Food Chem..

[21] Azevedo M.S., Tischer Seraglio S.K., Rocha G., Balderas C.B., Piovezan M., Gonzaga L.V., Falkenberg D.d.B., Fett R., Leal de Oliveira M.A., Oliveira Costa A.C. (2017). Free amino acid determination by GC-MS combined with a chemometric approach for geographical classification of bracatinga honeydew honey (Mimosa scabrella Bentham). Food Control.

[22] Chen H., Jin L., Chang Q., Peng T., Hu X., Fan C., Pang G., Lu M., Wang W. (2017). Discrimination of botanical origins for Chinese honey according to free amino acids content by high-performance liquid chromatography with fluorescence detection with chemometric approaches. J. Sci. Food Agric..

[23] Lerma-Garcia M.J., Ramis-Ramos G., Herrero-Martinez J.M., Simo-Alfonso E.F. (2007). Classification of vegetable oils according to their botanical origin using amino acid profiles established by direct infusion mass spectrometry. Rapid Commun. Mass Spectrom..

[24] Concha-Herrera V., Lerma-Garcia M.J., Herrero-Martinez J.M., Simo-Alfonso E.F. (2010). Classification of vegetable oils according to their botanical origin using amino acid profiles established by High Performance Liquid Chromatography with UV-vis detection: A first approach. Food Chem..

[25] Li L.-Q., Baibado J.T., Shen Q., Cheung H.-Y. (2017). Determination of the authenticity of plastron-derived functional foods based on amino acid profiles analysed by MEKC. J. Chromatogr. B.

[26] Lima M.J.R., Santos A.O., Falcao S., Fontes L., Teixeira-Lemos E., Vilas-Boas M., Veloso A.C.A., Peres A.M. (2019). Serra da Estrela cheese’s free amino acids profiles by UPLC-DAD-MS/MS and their application for cheese origin assessment. Food Res. Int..

[27] Association of Official Analytical Chemists (2006). Official Methods of Analysis.

[28] Cao W., Zhang C., Hong P., Ji H., Hao J., Zhang J. (2009). Autolysis of shrimp head by gradual temperature and nutritional quality of the resulting hydrolysate. LWT-Food Sci. Technol..

[29] Song G.S., Chen K., Wang H.X., Zhang M.N., Yu X.N., Wang J., Shen Q. (2020). In situ and real-time authentication of Thunnus species by iKnife rapid evaporative ionization mass spectrometry based lipidomics without sample pretreatment. Food Chem..

[30] Fiamegos Y., Dumitrascu C., Papoci S., de la Calle M.B. (2021). Authentication of PDO paprika powder (Pimenton de la Vera) by multivariate analysis of the elemental fingerprint determined by ED-XRF. A feasibility study. Food Control.

[31] Linehan L.G., O’Connor T.P., Burnell G. (1999). Seasonal variation in the chemical composition and fatty acid profile of Pacific oysters (*Crassostrea gigas*). Food Chem..

[32] Chen D.-W., Su J., Liu X.-L., Yan D.-M., Lin Y., Jiang W.-M., Chen X.-H. (2012). Amino acid profiles of bivalve mollusks from Beibu Gulf, China. J. Aquat. Food Prod. Technol..

[33] Chen Y.C., Tou J.C., Jaczynski J. (2009). Amino acid and mineral composition of protein and other components and their recovery yields from whole Antarctic krill (*Euphausia superba*) using isoelectric solubilization/precipitation. J. Food Sci..

[34] Wen J., Hu C., Fan S. (2010). Chemical composition and nutritional quality of sea cucumbers. J. Sci. Food Agric..

[35] Saito M., Kunisaki N., Urano N., Kimura S. (2002). Collagen as the major edible component of sea cucumber (*Stichopus japonicus*). J. Food Sci..

[36] Sadowska M., Kolodziejska I., Niecikowska C. (2003). Isolation of collagen from the skins of Baltic cod (*Gadus morhua*). Food Chem..

[37] Suzuki T., Shibata N. (1990). The utilization of Antarctic krill for human food. Food Rev. Int..

[38] Zhang J., Zheng H., Zhang C., Hao J., Zhang J., Zhang J. (2013). The separation and composition of Oyster protein. Food Ferment. Ind..

[39] Seow E.-K., Ibrahim B., Muhammad S.A., Lee L.H., Cheng L.-H. (2016). Differentiation between house and cave edible bird’s nests by chemometric analysis of amino acid composition data. LWT-Food Sci. Technol..

[40] Hajimahmoodi M., Vander Heyden Y., Sadeghi N., Jannat B., Oveisi M.R., Shahbazian S. (2005). Gas-chromatographic fatty-acid fingerprints and partial least squares modeling as a basis for the simultaneous determination of edible oil mixtures. Talanta.

[41] Cecchi L., Migliorini M., Giambanelli E., Rossetti A., Cane A., Mulinacci N., Melani F. (2020). Authentication of the geographical origin of virgin olive oils from the main worldwide producing countries: A new combination of HS-SPME-GC-MS analysis of volatile compounds and chemometrics applied to 1217 samples. Food Control.

[42] Nicolaou N., Xu Y., Goodacre R. (2010). Fourier transform infrared spectroscopy and multivariate analysis for the detection and quantification of different milk species. J. Dairy Sci..

