# Supplementary material for: Amino Acid Profiling with Chemometric Analysis as a Feasible Tool for the Discrimination of Marine-Derived Peptide Powders
Source: Foods. 2021 Jun 4;10(6):1294. doi: 10.3390/foods10061294 (PMC8229220; doi:10.3390/foods10061294)
Supplement: Supplementary file 1 [file foods-10-01294-s001.zip › foods-1235112-SI.pdf]

Table S1

Detailed information of the raw marine material samples

Sample	Species	Geographical location	Pre-treatment process	Sample Code
Oyster	/	Weihai (China)	Frozen	OM1-3
	/	Yantai (China)	Fresh	OM4-6
	/	Qingdao (China)	Fresh	OM7-9
	/	Dalian (China)	Frozen	OM10-12
Antarctic krill	*Euphausia superba*	/	Defatted meal	AK1-3
	Euphausia superba	/	Non-defatted meal	AK4-6
	Euphausia superba	/	Frozen	AK7-9
	Euphausia superba	/	Shelled and frozen	AK10-12
Fish skin	Cod	/	Frozen	FS1-3
	Tilapia	/	Frozen	FS4-6
Sea cucumber	*Acaudina molpadioides*	/	Dried and boiled	SC1-3
	Acaudina molpadioides	/	Dried	SC4-5
	Apostichopus japonnicus	/	Salted and boiled	SC6
	Apostichopus japonnicus	/	Salted	SC7
	Holotoria floridona	/	Dried	SC8
	Holothuria hilla	/	Dried	SC9
	Cucumaria frondosa	/	Dried	SC10
	Cucumaria frondosa	/	Frozen and boiled	SC11
	Cucumaria frondosa	/	Frozen	SC12

Table S2

Crude protein content and hydrolyzed amino acid composition of nine unclassified peptide samples

Amino acid[a]	Amino acid content (g/100g dry protein basis)								
	T1[b]	T2[b]	T3[b]	T4[b]	T5[b]	T6[b]	T7[b]	T8[b]	T9[b]
TAU	0.73	11.31	5.61	3.05	0.74	0.78	15.10	0.00	0.00
ASP	14.72	8.64	8.64	10.21	8.19	8.21	13.18	1.75	6.40
THR	5.69	3.77	3.69	4.23	3.75	3.73	6.08	1.15	2.02
SER	4.62	4.61	3.83	4.24	3.56	3.44	6.16	1.17	3.53
GLU	13.55	13.85	13.04	15.29	14.50	14.50	19.78	2.99	10.96
GLY	7.02	8.18	5.65	7.34	6.00	5.92	7.85	22.57	24.89
ALA	4.47	5.58	4.77	5.99	5.61	5.35	6.94	18.35	9.82
CYS	0.67	0.00	0.00	0.00	0.00	0.00	0.00	0.61	0.00
VAL	4.89	4.41	3.77	4.45	4.00	3.87	6.43	14.71	3.51
MET	2.19	2.43	2.11	2.39	2.11	2.08	3.15	0.20	1.46
ILE	3.80	3.33	3.55	3.92	3.55	3.46	5.63	3.11	1.79
LEU	4.42	5.93	6.36	7.46	6.52	6.60	9.85	7.73	3.18
TYR	2.57	2.84	2.85	3.22	2.74	2.72	4.74	2.27	1.16
PHE	2.63	2.59	2.44	2.56	2.45	2.44	3.51	5.23	2.19
LYS	4.53	5.11	6.21	7.15	8.14	8.10	9.66	0.98	3.77
HIS	1.97	1.16	1.49	1.83	2.35	2.31	2.60	0.24	0.79
ARG	4.19	10.24	6.92	8.26	5.94	5.90	10.79	1.16	8.20
PRO	6.93	6.60	3.11	3.78	3.89	3.84	4.77	9.42	14.55
Total AAs (g/100g dry protein basis)	65.71	82.53	74.60	84.65	80.76	79.98	98.39	87.04	84.08
Crude Protein (g/100g dry sample basis)	69.06	77.24	83.58	83.58	90.45	90.45	68.00	87.50	80.59

[a], Abbreviation: TAU, taurine; ASP, aspartic acid; THR, threonine; SER, serine; GLU, glutamic acid; GLY, glycine; ALA, alanine; CYS, cysteine; VAL, valine; MET, methionine; ILE, isoleucine; LEU, leucine; TYR, tyrosine; PHE, phenylalanine; LYS, lysine; HIS, histidine; ARG, arginine; PRO, proline; Total AAs, total amino acids.

[b], T1-2: Abalone peptides; T3-4: Octopus peptides; T5-6: Crocodile peptides; T7-Cuttlefish peptides; T8: bull backstrap peptides; T9: Donkey hides gelatin peptides.
